# PRID: Prediction Model Using RWR for Interactions between Drugs

**DOI:** 10.3390/pharmaceutics15102469

**Published:** 2023-10-15

**Authors:** Jiwon Seo, Hyein Jung, Younhee Ko

**Affiliations:** Division of Biomedical Engineering, Hankuk University of Foreign Studies, Yongin 17035, Gyeonggi-do, Republic of Korea; jiwons7500@hufs.ac.kr (J.S.); fff0992@hufs.ac.kr (H.J.)

**Keywords:** drug–drug interaction, RWR, deep learning

## Abstract

Drug–drug interactions (DDI) occur because of the unexpected pharmacological effects of drug pairs. Although drug efficacy can be improved by taking two or more drugs in the short term, this may cause inevitable side effects. Currently, multiple drugs are prescribed based on the experience or knowledge of the clinician, and there is no standard database that can be referred to as safe co-prescriptions. Thus, accurately identifying DDI is critical for patient safety and treatment modalities. Many computational methods have been developed to predict DDIs based on chemical structures or biological features, such as target genes or functional mechanisms. However, some features are only available for certain drugs, and their pathological mechanisms cannot be fully employed to predict DDIs by considering the direct overlap of target genes. In this study, we propose a novel deep learning model to predict DDIs by utilizing chemical structure similarity and protein–protein interaction (PPI) information among drug-binding proteins, such as carriers, transporters, enzymes, and targets (CTET) proteins. We applied the random walk with restart (RWR) algorithm to propagate drug CTET proteins across a PPI network derived from the STRING database, which will lead to the successful incorporation of the hidden biological mechanisms between CTET proteins and disease-associated genes. We confirmed that the RWR propagation of CTET proteins helps predict DDIs by utilizing indirectly co-regulated biological mechanisms. Our method identified the known DDIs between clinically proven epilepsy drugs. Our results demonstrated the effectiveness of PRID in predicting DDIs in known drug combinations as well as unknown drug pairs. PRID could be helpful in identifying novel DDIs and associated pharmacological mechanisms to cause the DDIs.

## 1. Introduction

The efficacy of a drug can be affected by various factors, such as dosage, ingestion method, genetic background, and drug interactions. Prescription of two or more drugs is sometimes necessary to clinically control the complex symptoms. Although taking multiple drugs for a short period can increase efficacy, unavoidable side effects could occur with chronic usage. Especially, drug–drug interactions (DDI) result from the unexpected pharmacological effects of drug pairs.

An adverse drug event (ADE) is an unwanted occurrence after exposure to a drug. The number of reported ADEs is increasing, affecting approximately 20,000,000 patients annually. Drug–drug interactions account for 30% of ADEs, which is one-third of all adverse events in the United States [1,2]. With increased drug usage, the probability of patients developing ADE also increases. According to the U.S. Centers for Disease Control and Prevention, 20% of older adults simultaneously take ten or more medications [3]. It is essential to be aware of DDIs and avoid prescribing drugs in advance to prevent ADEs caused by DDI. However, due to the practical infeasibility of testing all conceivable drug–drug interactions, the majority of drug–drug interactions remain undiscovered.

Developing new drugs is time-consuming and costly [4], but even those verified by in vivo and in vitro experiments are often withdrawn from the market owing to unexpected side effects. Thus, predicting potential DDIs among new or existing drugs has become critical. Recently, many computational methods [5,6,7] have been applied to predict potential DDIs through the integration of heterogeneous drug-related data (e.g., chemical structure [7,8,9]; target proteins [10]; expression profile; or side effects). However, it is very challenging to extract the features of the input data and transform them into suitable forms for a model because of data inconsistencies and missing problems through the integration of heterogeneous data [11,12]. As various types of data, such as the chemical structure of drugs, drug targets, and the side effects of drugs, become available, many studies [5,6,7,8,9,10] have been employed to identify the interactions of drugs, encompassing similarity-based approaches [6,13,14,15], such as leveraging drug profiles including pharmaceutical profiles, gene expression profiles or phenotypic data. Nevertheless, determining the appropriate metrics for measuring the similarity between these drug profiles remains a challenging task. Network propagation-based approaches [11,16,17,18] were applied to predict the links between drugs within the biological network by incorporating the network structure. Additionally, recent endeavors have incorporated the matrix factorization method [7,19] to decompose the drug interaction matrix to predict drug interactions. Ensemble-based approaches [20,21,22] have emerged to combine multiple methods, leading to improved prediction accuracy. Deep learning models [5,23] have been effectively applied to integrate large amounts of heterogeneous data. While these approaches can be useful to identify DDIs caused by common direct pathological mechanisms, they cannot predict the DDIs occurred by indirectly shared pathways.

**Figure 1:**
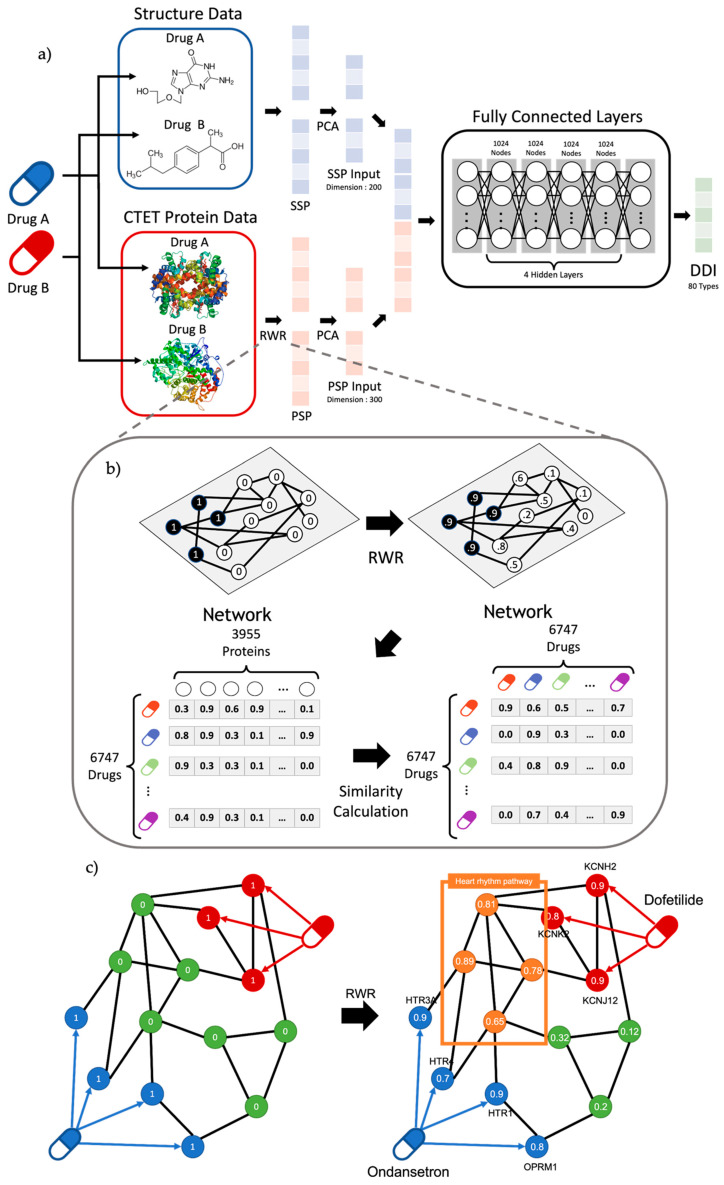
(**a**) Overview of workflow. The model uses transformed structure data and CTET protein data called SSP and PSP. After dimension reduction of SSP and PSP with PCA, the model was trained to classify 80 DDI types. Our model comprises six layers, including the input/output layer with 1024 nodes for hidden layers. (**b**) Overview of RWR algorithm and PSP calculation. RWR algorithm propagates the effect of seed nodes by setting CTET protein as starting nodes. Based on the propagated result of RWR, the PSP profile of a drug was calculated with the similarities between a drug and the rest of the drugs. Note that the similar propagated result among proteins (i.e., PSP profile) represents similar pharmacological effects. (**c**) Graph figure about DDI between Ondansetron and Dofetilide. Results of RWR show that target genes can affect proteins at the heart rhythm pathway.

In this study, we propose a novel deep learning model to predict DDIs based on the chemical structure of drugs, CTET proteins related to drug processing, and their hidden associations embedded in a protein–protein interaction (PPI) network (Figure 1). In particular, the pharmacologically associated hidden mechanisms have been identified using the Random Walk with Restart (RWR) algorithm [24] and have been successfully applied to predict the hidden biological mechanisms associated with DDIs. Unlike traditional DDI prediction methods [7,12,25], our model (PRID) effectively predicted 80 DDI interaction types, and its accuracy was significantly higher than that of other methods. Novel interactions between the new drugs were successfully predicted and confirmed through a literature review.

## 2. Results

### 2.1. Performance of PRID Model

**Figure 2:**
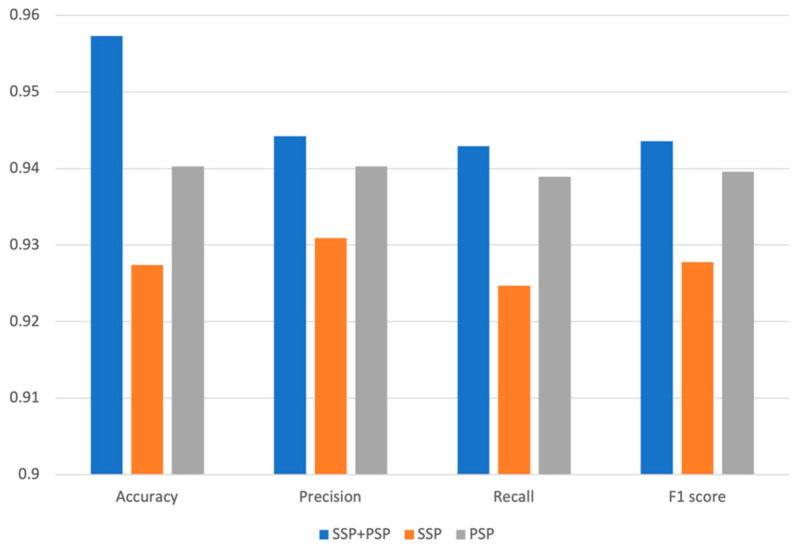
Accuracy, precision, recall, and F1 of models trained by only SSP, only PSP, and both SSP and PSP.

To identify the biological significance of the various features for predicting DDIs, we compared the prediction performances of three different models using different features, such as SSP, PSP, and SSP + PSP. We have partitioned our dataset into a training set and a test set in a 7:3 ratio while ensuring an equitable distribution of each DDI type. The model was subsequently trained using the training set, and its performance was evaluated with the test set. To assess the validity and predictivity of the model, we employed a 4-fold cross-validation strategy. The rationale behind selecting 4-fold cross-validation was to ensure an even distribution of interactions across each fold. The smallest number of interactions within a fold was 4, and to accommodate this, we chose 4-fold cross-validation. We observed consistently high accuracy levels in validation across all folds, and the accuracy for different interaction types was also uniformly high, with no significant variation between folds. The chemical structure similarity profile of the drug (SSP) and the protein structure similarity profile (PSP) itself, are critical for predicting DDI types; however, when combined together, the overall performance of our model was dramatically improved. The model’s accuracy using both SSP and PSP was 0.957; however, individually, SSP and PSP yielded 0.929 and 0.927 accuracy, respectively [7,8,9]. The superior performance of our DNN model using both SSP and PSP was observed for all other metrics, such as precision, recall, and F1 score (Figure 2). PRID is a multi-label classification model. To perform performance evaluation, we calculate precision, recall, and F1 score for each interaction type label separately and then compute the macro-precision and macro-recall by taking the averages. The F1 scores are calculated using the precision and recall values obtained.

**Figure 3:**
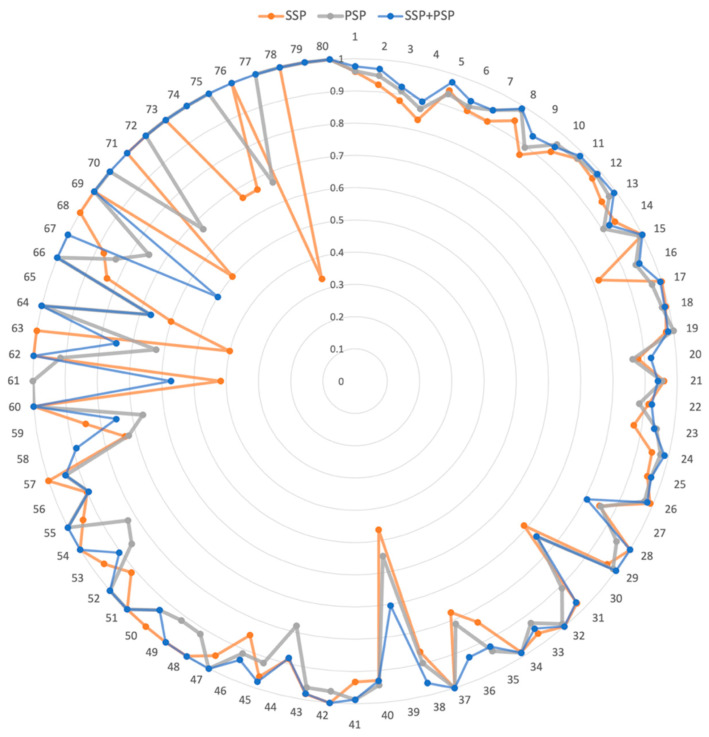
The radar graph for accuracy of 80 DDI types. SSP, PSP, and SSP + PSP represent the model using drug structure information, drug CTET protein information, and both, respectively. Each DDI type was positioned at an angle, and the distance from the center indicated the accuracy of each DDI type.

We further investigated the prediction accuracy of individual DDI interaction types (Figure 3). The DDI numbers were ordered by the number of drug pairs belonging to the DDI type (Supplementary Table S1). For example, DDI Type 1 was the most prevalent interaction type, with 60,997 drug pairs. In the radar graph (Figure 3), the higher the accuracy, the farther the point was from the position. Thus, the resultant graph shape was a circle when the model accurately performed on all interactions. Overall, the model using both SSP and PSP performed better than those using only SSP or PSP. Our model was more accurate than the others, especially for the 68–79 DDI types. Notably, 68–79 DDI types had a relatively small number of drug pairs. Thus, we demonstrated that the combination of SSP and PSP in our model dramatically improved the accuracy as well as effectively predicted DDI types, especially with insufficient training data.

### 2.2. Clinical Validation for Predicted DDI

We applied our DNN model on clinically proven epilepsy drug pairs to confirm its predictive performance (Table 1) [26]. Of the 48 known epilepsy drug pairs, 40 were already included in our training data, and the remaining 8 drug pairs were tested. Our DNN model identified known DDI between epilepsy drug pairs (Table 1). When lamotrigine and acetaminophen, or diazepam and ciprofloxacin, are combined, the risk or severity of adverse effects increases (DDI type 1). Our DNN model could effectively identify known DDI types. Furthermore, the co-prescription of valproic acid and acyclovir is also known to reduce the bioavailability of acyclovir [27] and our model could successfully predict such drug interactions (Table 1). In addition to general interaction types, such as DDI type 1, more specific drug–drug interactions, such as DDI type 8 or 45, have been identified. For example, Clonazepam is known to have severe adverse effects, such as excessive drowsiness, confusion, and difficulty concentrating when taken with Cimentidine or Omoprazole. Our model successfully predicts their interactions as DDI type 8: “Drug a may increase the central nervous system depressant (CNS depressant) activities of Drug b”.

### 2.3. Validation for False Positive Data

Among the 80 DDI types, some types of DDI are relatively similar or opposite to one another (Table 2). For instance, DDI type 1 is annotated as “The risk or severity of adverse effects can be increased when Drug A is combined with Drug B”, and DDI type 9 is explained as “The metabolism of Drug B can be increased when combined with Drug A”. It is evident that type 9 provides a more detailed description than type 1 for DDI. If the model predicts a DDI of type 1 as type 9, the prediction is classified as false. However, this prediction may be accurate in the real world. These two drugs can affect each other through drug metabolism, and our methods can confirm the more specific consequences of DDI. Therefore, we further investigated interactions classified as false positives using the proposed algorithm. First, we sorted all potential false-positive interactions according to the confidence scores. Since a high confidence score represents strong evidence for such interactions, we took a closer look at the top 10 high-scoring DDI based on our DDN model (Table 2). Pentobarbital and phenobarbital are barbiturates used to treat insomnia. DrugBank indicates DDI for the two drugs as type 1. However, in our method, PRID predicted DDI for those two drugs as type 9, “The serum concentration of the active metabolites of Drug B can be increased when Drug B is used in combination with Drug A”. Since both drugs are designed for the same pharmacological mechanism, their co-prescription leads to increased drug metabolism, and our method could identify more specific side effects of drug combinations. Many false-positive DDI pairs were confirmed through a literature review. The combination of griseofulvin and alcohol was also predicted to be a DDI type 9, suggesting it may increase absorption [26]. Additionally, Mifepristone and Bedaquiline increase QTc prolonging activities, occasionally resulting in abnormal heart rhythms [26].

### 2.4. Comparison of RWR Methods and Non-RWR Methods

RWR performs random walks based on connections between nodes, and while probabilistically moving from a specific node to other nodes. By incorporating the RWR algorithm into the PRID model, utilizing CTET proteins as seed nodes in the protein–protein interaction network, we can effectively leverage not only the proteins that have direct interaction with drugs but also those proteins that exhibit indirect interaction with drugs. This approach enables the identification of intricate drug–drug interactions.

**Figure 4:**
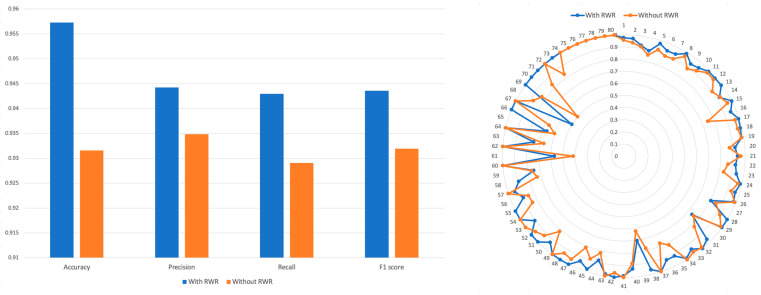
Comparison of accuracy, precision, recall, and F1 of models trained by both SSP and RWR-generated PSP. The performance of model was evaluated when the PRID model is trained with RWR or without RWR.

We calculated the Tanimoto similarity among CTET proteins of drugs and created a PSP without utilizing the RWR algorithm. A binary vector representing the CTET proteins was created, by marking the CTET proteins of each drug as 1. Then, Tanimoto coefficients were computed between these binary vectors to generate the PSP profile without using RWR. We then concatenated this with SSP and used it for training (see Methods Section). The results (Figure 4) showed that the model trained using the RWR-generated PSP achieved an accuracy of 0.957, while the model trained using the non-RWR-generated PSP achieved an accuracy of 0.931. Precision, recall, and F1 scores were all higher in the model trained using the RWR-generated PSP compared to the model trained using the non-RWR-generated PSP.

## 3. Discussion

Identifying hidden DDIs is crucial to reducing the potential adverse drug effects due to the co-prescription of drugs. However, experimental identification of all the existing drug interactions is not feasible. Instead, drug–drug interactions can be identified with computational methods by utilizing various drug features, such as chemical structure, target genes, or associated pathological mechanisms. DDIs typically result from unexpected associations between target proteins or indirect biologically shared mechanisms. Identifying DDIs is challenging without considering such interference associations. Thus, we utilized the hidden pharmacological mechanisms associated with the CTET proteins of drug pairs to identify DDIs. These hidden pharmacological mechanisms were incorporated into our model using the RWR algorithm to handle indirect PPIs. We proposed a DNN model to predict DDIs based on chemical structure similarities and shared biological mechanisms through the CTET proteins of drugs. The RWR algorithm effectively incorporates indirectly connected or affected protein interactions into the model, and our model predicts the DDI types for drug pairs with the highest confidence scores. However, some drug pairs do not exhibit such interactions. Thus, we assigned a DDI for drug pairs when the highest confidence score was sufficiently large, and no interaction was predicted unless the highest confidence score of each drug pair exceeded the threshold. The threshold for DDI assignment was set at 0.47, which was empirically determined based on prediction accuracy (Supplementary Figure S1). Notably, determining whether such non-interacting drug pairs have real drug interactions or whether current information is sufficient to predict the drug pair interactions was challenging. These drug pairs can be further investigated to validate their associations when additional data becomes available and predicted DDIs which are unknown, can be experimentally tested for clinical validation. However, there are still obstacles we have to overcome. Although there are computationally predicted DDIs with strong confidence, in order to prescribe to the patients, drug pairs without DDIs must be demonstrated to be clinically safe.

In conclusion, we proposed a novel algorithm to predict drug–drug interactions by incorporating the chemical structure of drugs and their shared pharmacological mechanisms through the RWR algorithm. Even for drug pairs that were not directly related, we successfully identified potential DDIs by considering the common hidden mechanisms between drugs. Our method can be applied to all drug pairs, and the predicted potential DDI can be used clinically with experimental validations, leading to drug repurposing.

## 4. Methods

### 4.1. Drug–Drug Interaction Data

DrugBank [28] is a public online database containing information on drugs and drug targets or DDIs. We downloaded 192,284 Food and Drug Administration-approved drug interaction data from this database. Drug–drug interactions were described in the form of sentences, and 86 DDIs were extracted. Among the 86 interaction types, 6 with a relatively small number of DDIs were removed, and 80 interaction types were used in our study, leading to 171,280 DDIs covering 1710 drugs (Supplementary Table S1). Among the 1710 drugs, only 1436 had information on chemical structure and CTET proteins and were used to predict drug pairs. Consequently, 163,574 DDIs were used for model training and testing.

### 4.2. Protein–Protein Interaction Network

STRING is a database of known and predicted PPIs. These interactions include direct (physical) and indirect (functional) associations, which are extracted from computational predictions, interaction transfers between organisms, or other databases. It contained 20,772 proteins with Ensembl protein identifiers and 2,425,315 interactions. Since Entrez identifiers represented our gene sets, the Ensembl protein identifiers in the original STRING database were converted to Entrez identifiers using the mapping information in the STRING database. This resulted in 18,074 genes with 2,153,757 interactions.

### 4.3. SSP (Structure Similarity Profile) of the Drug

Each drug has a unique chemical structure. These structures are critical features for identifying DDIs. The molecular structures of 2460 FDA-approved drugs were downloaded from DrugBank. First, the chemical structure of each drug in the SMILES (Simplified Molecular-input Line-entry System) format was transformed into a binary numerical vector. RDKit was employed to convert the chemical structure fingerprints into a binary vector format. Each value in the binary vector represents whether the drug possesses a specific chemical structure. The structural similarity (SS) between these two drug pairs was calculated using the Tanimoto coefficient, which is defined as the proportion of common structures. The SSP of each drug was defined as a vector of SSs by combining the SS over all the other drugs, the size of which was the total number of drugs. Thus, the SSP vector of each drug represented its chemical structure feature and contained the pairwise structure similarity scores based on the comparison with all the other drugs (Figure 1).

### 4.4. PSP (Protein Similarity Profile) of the Drug and RWR

Drugs are processed in the human body, and many proteins or enzymes, such as CTET proteins, are usually involved in the absorption, distribution, metabolism, and excretion of these drugs. In this study, we compiled CTET proteins from DrugBank, covering 6747 drugs and 3955 CTET proteins. Drug–drug interactions occur when the pharmacological effects of drugs interact with each other. We used functional relationships between CTET proteins and the biological pathways associated with them to identify DDIs. However, most drugs have a relatively small number of CTET proteins, and such drug pairs do not directly share a common CTET protein; instead, they have an indirectly shared mechanism leading to DDIs. Therefore, we attempted to identify the hidden pharmacological mechanisms between the two drugs to predict DDIs using the RWR algorithm. This algorithm is widely used to measure the association between vertices of the graph by considering their connectivity through the network.

The equation of RWR can be defined as Equation (1):

(1) $$p\left(t+1\right)=\left(1-r\right)M^{T}p\left(t\right)+rp\left(0\right)$$

*p*(*t*) represents the probability vector of each node at time step *t*, and *p*(0) represents the initial probability vector, where *M* denotes the normalized adjacency matrix of the network. At each iteration, the value of *p*(*t*) is randomly propagated into the network or restarted from *p*(0) with restart probability *r*. We adopted the RWR algorithm to identify the structural closeness of CTET proteins between the two drugs in the PPI network (Figure 1). A PPI network was constructed from the STRING database [29] and only PPI interactions with the top 20% confidence score were extracted, including CTET proteins, which led to 5,568,534 interactions. The RWR algorithm was then applied to the PPI network to identify the hidden associations between the CTET proteins of the drugs. The association score between drugs A and B was calculated as an average of the propagated values for CTET proteins of Drug B when the CTET proteins of drugs were used as seed genes in the RWR algorithm.

Similarly, the RWR algorithm was applied to all other drugs in the database, given Drug A, and the obtained association scores were represented as a protein similarity profile (PSP) vector of a given drug (Figure 1b). The RWR algorithm allows us to consider the hidden biological pathways associated with the mechanism of drugs to predict drug interactions. For example, the co-prescription of Dofetilide and Ondansetron can lead to bradycardia or a slower heart rate, resulting in severe consequences for patients. Although these two drugs do not directly target the heart rhythm-associated pathway, they can be indirectly affected by these drugs, which were successfully incorporated to identify unexpected DDIs through the RWR algorithm (Figure 1c).

### 4.5. Deep Neural Network Structure for Prediction of DDIs

We compiled two heterogeneous datasets (molecular drug structure and CTET proteins of the drug), and the SSP and PSP feature vectors were constructed as input data for our multilayer perceptron (MLP) model to predict DDIs. We applied principal component analysis (PCA) to reduce the dimensions of the SSP and PSP vectors, resulting in 200 dimensions for SSP and 300 dimensions for PSP (Figure 1a) because of their high-dimensional nature. We explored several model structures with different numbers of hidden layers and nodes (Supplementary Figure S1), and the final structure with the best performance had four hidden layers with 1024 nodes (Figure 1a). We set the batch size to 128 and the learning rate to 0.001. The number of epochs was set to 100, and the dropout rate was set to 0.3. The optimizer was Adam, and the activation function was ReLU. The DDI with the highest probability (e.g., confidence score) was predicted to be the designated DDI type. We set the decision threshold for the DDI prediction to 0.47, which was determined using the F1 score (Supplementary Figure S2). Therefore, a drug–drug pair with a probability greater than a predefined threshold is predicted to be of the specific DDI type among 80 DDI types. Drug–drug interaction types compiled from DrugBank were used as the gold standard for training and testing, and clinical epilepsy drug pairs were used for further validation.

## Figures and Tables

**Table 1:** The predicted DDI types between epilepsy drugs and the other drugs. Drugbank ID and name of drug of the drug pairs, predicted DDI type, and the confidence score of the model are shown in order.

Drug A	Drug Name 1	Drug B	Drug Name 2	Interaction	Confidence
DB00555	Lamotrigine	DB00316	Acetaminophen	1	1
DB00829	Diazepam	DB00537	Ciprofloxacin	1	1
DB00313	Valproic acid	DB00787	Acyclovir	1	0.999995589
DB01068	Clonazepam	DB00501	Cimetidine	8	0.999974012
DB01068	Clonazepam	DB00338	Omeprazole	8	0.966016471
DB00313	Valproic acid	DB00199	Erythromycin	45	0.768196225
DB01068	Clonazepam	DB00951	Isoniazid	1	0.558177412
DB00252	Phenytoin	DB00364	Sucralfate	4	0.476196706

1: The risk or severity of adverse effects can be increased when Drug A is combined with Drug B. 4: Drug A may increase the hypotensive activities of Drug B. 8: Drug A may increase the central nervous system depressant (CNS depressant) activities of Drug B. 45: The serum concentration of the active metabolites of Drug B can be reduced when Drug B is used in combination with Drug A, resulting in the loss of efficacy.

**Table 2:** Top 10 false positive results of our model and description for predicted DDIs.

Drug A	Drug A Name	Drug B	Drug B Name	Predict	Label	Prediction Sentence
DB00312	Pentobarbital	DB01174	Phenobarbital	9	1	The metabolism of Drug B can be increased when combined with Drug A.
DB00215	Citalopram	DB06589	Pazopanib	2	3	The metabolism of Drug B can be decreased when combined with Drug A.
DB00349	Clobazam	DB00734	Risperidone	5	2	Drug A may increase the hypotensive activities of Drug B.
DB08881	Vemurafenib	DB00972	Azelastine	4	3	The serum concentration of Drug B can be decreased when combined with Drug A.
DB00400	Griseofulvin	DB00898	Ethanol	9	1	The metabolism of Drug B can be increased when combined with Drug A.
DB01045	Rifampicin	DB00199	Erythromycin	27	9	The serum concentration of the active metabolites of Drug B can be increased when Drug B is used in combination with Drug A.
DB01174	Phenobarbital	DB00541	Vincristine	9	4	The metabolism of Drug B can be increased when combined with Drug A.
DB01026	Ketoconazole	DB12001	Abemaciclib	2	3	The metabolism of Drug B can be decreased when combined with Drug A.
DB00834	Mifepristone	DB08903	Bedaquiline	7	3	The risk or severity of QTc prolongation can be increased when Drug A is combined with Drug B.
DB01104	Sertraline	DB01418	Acenocoumarol	10	2	Drug A may increase the anticoagulant activities of Drug B.

## Data Availability

The datasets used and/or analyzed during the current study are available from the corresponding author on reasonable request and the rest of the data that has been used are provided in the Supplementary Files.

## Supplementary Materials

The following supporting information can be downloaded at: https://www.mdpi.com/article/10.3390/pharmaceutics15102469/s1, Figure S1: Graph of model’s accuracy depend on number of hidden layer and number of node in hidden layer; Figure S2: Graph of F1 score for decision threshold; Table S1: DDI types, descriptions and number of DDI.
