# Supplementary material for: PRID: Prediction Model Using RWR for Interactions between Drugs
Source: Pharmaceutics. 2023 Oct 15;15(10):2469. doi: 10.3390/pharmaceutics15102469 (PMC10610536; doi:10.3390/pharmaceutics15102469)

Supplementary 1. DDI types, descriptions and number of DDI

DDI Type	Description	DDI Number
**1**	The risk or severity of adverse effects can be increased when Drug a is combined with Drug b.	60997
**2**	The metabolism of Drug b can be decreased when combined with Drug a.	34360
**3**	The serum concentration of Drug b can be increased when it is combined with Drug a.	23779
**4**	The serum concentration of Drug b can be decreased when it is combined with Drug a.	9506
**5**	Drug a may increase the hypotensive activities of Drug b.	8397
**6**	The therapeutic efficacy of Drug b can be decreased when used in combination with Drug a.	7788
**7**	Drug a may increase the QTc-prolonging activities of Drug b.	6140
**8**	Drug a may increase the central nervous system depressant (CNS depressant) activities of Drug b.	5435
**9**	The metabolism of Drug b can be increased when combined with Drug a.	5011
**10**	Drug a may increase the anticoagulant activities of Drug b.	3160
**11**	Drug a may decrease the antihypertensive activities of Drug b.	3089
**12**	Drug a may increase the hypoglycemic activities of Drug b.	2109
**13**	Drug a may decrease the excretion rate of Drug b which could result in a higher serum level.	1825
**14**	Drug a may increase the bradycardic activities of Drug b.	1277
**15**	Drug a may increase the hypokalemic activities of Drug b.	1204
**16**	Drug a can cause a decrease in the absorption of Drug b resulting in a reduced serum concentration and potentially a decrease in efficacy.	1092
**17**	Drug a may decrease the cardiotoxic activities of Drug b.	1043
**18**	Drug a may increase the sedative activities of Drug b.	1015
**19**	Drug a may increase the neuroexcitatory activities of Drug b.	936
**20**	Drug a may increase the serotonergic activities of Drug b.	803
**21**	Drug a may increase the atrioventricular blocking (AV block) activities of Drug b.	716
**22**	Drug a may increase the hypertensive activities of Drug b.	673
**23**	Drug a may increase the nephrotoxic activities of Drug b.	664
**24**	Drug a may increase the antihypertensive activities of Drug b.	629
**25**	Drug a may increase the orthostatic hypotensive activities of Drug b.	616
**26**	Drug a may decrease the sedative activities of Drug b.	558
**27**	The serum concentration of the active metabolites of Drug b can be increased when Drug b is used in combination with Drug a.	538
**28**	The bioavailability of Drug b can be decreased when combined with Drug a.	519
**29**	Drug a may decrease the stimulatory activities of Drug b.	498
**30**	The risk or severity of QTc prolongation can be increased when Drug a is combined with Drug b.	443
**31**	Drug a may increase the neuromuscular blocking activities of Drug b.	428
**32**	Drug a may increase the fluid retaining activities of Drug b.	422
**33**	Drug a may increase the tachycardic activities of Drug b.	372
**34**	Drug a may decrease the bronchodilatory activities of Drug b.	361
**35**	Drug a may increase the arrhythmogenic activities of Drug b.	355
**36**	Drug a may increase the antiplatelet activities of Drug b.	336
**37**	Drug a may decrease the diuretic activities of Drug b.	324

**38**	Drug a may increase the anticholinergic activities of Drug b.	323
**39**	The serum concentration of the active metabolites of Drug b can be reduced when Drug b is used in combination with Drug a resulting in a loss in efficacy.	313
**40**	Drug a may increase the immunosuppressive activities of Drug b.	312
**41**	Drug a may decrease the vasoconstricting activities of Drug b.	309
**42**	Drug a may increase the respiratory depressant activities of Drug b.	300
**43**	Drug a may increase the analgesic activities of Drug b.	280
**44**	Drug a may increase the hyperkalemic activities of Drug b.	278
**45**	The therapeutic efficacy of Drug b can be increased when used in combination with Drug a.	245
**46**	Drug a may decrease the anticoagulant activities of Drug b.	238
**47**	Drug a may increase the cardiotoxic activities of Drug b.	202
**48**	Drug a may increase the hypocalcemic activities of Drug b.	180
**49**	Drug a may increase the constipating activities of Drug b.	148
**50**	The risk or severity of bleeding can be increased when Drug a is combined with Drug b.	128
**51**	Drug a may increase the hyponatremic activities of Drug b.	118
**52**	Drug a may increase the vasoconstricting activities of Drug b.	109
**53**	Drug a may increase the thrombogenic activities of Drug b.	108
**54**	Drug a may increase the antipsychotic activities of Drug b.	94
**55**	Drug a may increase the adverse neuromuscular activities of Drug b.	94
**56**	Drug a may decrease the neuromuscular blocking activities of Drug b.	83
**57**	Drug a may increase the hypercalcemic activities of Drug b.	83
**58**	Drug a can cause an increase in the absorption of Drug b resulting in an increased serum concentration and potentially a worsening of adverse effects.	82
**59**	Drug a may increase the myopathic rhabdomyolysis activities of Drug b.	69
**60**	Drug a may increase the vasopressor activities of Drug b.	65
**61**	Drug a may increase the hepatotoxic activities of Drug b.	64
**62**	Drug a may increase the stimulatory activities of Drug b.	56
**63**	The absorption of Drug b can be decreased when combined with Drug a.	45
**64**	Drug a may increase the ulcerogenic activities of Drug b.	43
**65**	Drug a may increase the myelosuppressive activities of Drug b.	34
**66**	Drug a may decrease effectiveness of Drug b as a diagnostic agent.	33
**67**	Drug a may increase the vasodilatory activities of Drug b.	33
**68**	The risk or severity of hypotension can be increased when Drug a is combined with Drug b.	33
**69**	Drug a may increase the hyperglycemic activities of Drug b.	28
**70**	Drug a may increase the central nervous system depressant (CNS depressant) and hypertensive activities of Drug b.	27
**71**	The risk of a hypersensitivity reaction to Drug b is increased when it is combined with Drug a.	27
**72**	Drug a may increase the bronchoconstrictory activities of Drug b.	26
**73**	The risk or severity of heart failure can be increased when Drug b is combined with Drug a.	26
**74**	Drug a may increase the hypotensive and central nervous system depressant (CNS depressant) activities of Drug b.	14

**75**	The risk or severity of hypertension can be increased when Drug b is combined with Drug a.	14
**76**	Drug a may increase the central neurotoxic activities of Drug b.	13
**77**	The protein binding of Drug b can be decreased when combined with Drug a.	11
**78**	The bioavailability of Drug b can be increased when combined with Drug a.	11
**79**	Drug a may increase the dermatologic adverse activities of Drug b.	11
**80**	Drug a may decrease the analgesic activities of Drug b.	10

Supplementary Figure 1. Graph of model's accuracy depend on number of hidden layer and number of node in hidden layer

	2	3	4	5	6
256	95.12979	94.869663	94.563995	94.414829	94.35614
512	95.718198	95.615495	95.451655	95.378295	95.278036
1024	95.713308	95.713308	95.73287	95.63313	95.366068

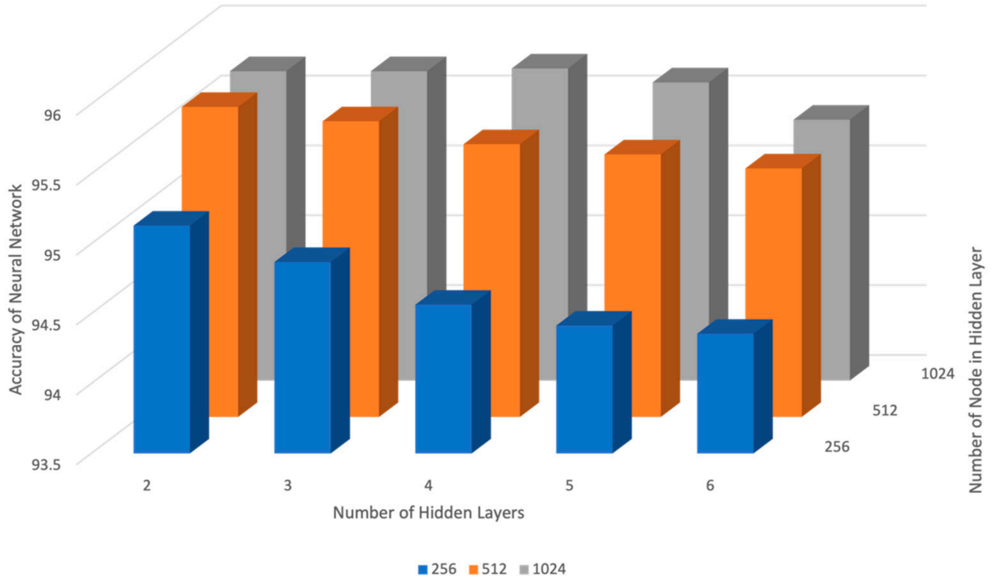

Supplementary Figure 2. Graph of F1 score for decision threshold

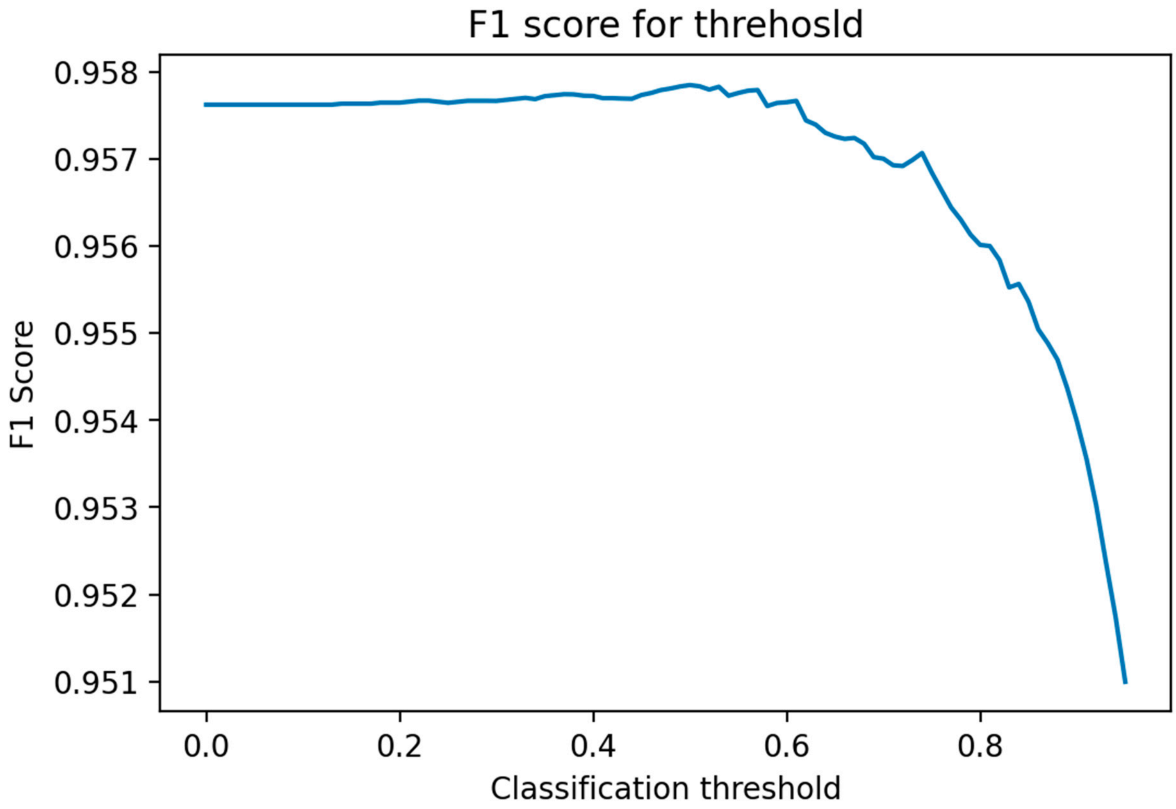

Supplement: Supplementary file 1 [file pharmaceutics-15-02469-s001.zip › pharmaceutics-2617050-supplementary.pdf]
